# Impact of the prognostic nutritional index on renal replacement therapy–free survival and mortality in patients on continuous renal replacement therapy

**DOI:** 10.1080/0886022X.2024.2365394

**Published:** 2024-06-14

**Authors:** Yu-Fu Lee, Pei-Ru Lin, Shin-Hwar Wu, Hsin-Hui Hsu, Shu-Yun Yang, Chew-Teng Kor

**Affiliations:** a Division of Critical Care Internal Medicine, Department of Emergency Medicine and Critical Care, Changhua Christian Hospital, Changhua, Taiwan; b Big Data Center, Changhua Christian Hospital, Changhua, Taiwan; c Graduate Institute of Statistics and Information Science, National Changhua University of Education, Changhua, Taiwan; d Graduate Institute of Clinical Medicine, College of Medicine, National Chung Hsing University, Taichung, Taiwan

**Keywords:** Continuous renal replacement therapy, prognostic nutritional index, renal replacement therapy–free survival, mortality, ICU, critically ill patient

## Abstract

**Background:**

The survival of critically ill patients with acute kidney injury (AKI) undergoing continuous renal replacement therapy (CRRT) is highly dependent on their nutritional status.

**Objectives:**

The prognostic nutritional index (PNI) is an indicator used to assess nutritional status and is calculated as: PNI = (serum albumin in g/dL) × 10 + (total lymphocyte count in/mm^3^) × 0.005. In this retrospective study, we investigated the correlation between this index and clinical outcomes in critically ill patients with AKI receiving CRRT.

**Methods:**

We analyzed data from 2076 critically ill patients admitted to the intensive care unit at Changhua Christian Hospital, a tertiary hospital in central Taiwan, between January 1, 2010, and April 30, 2021. All these patients met the inclusion criteria of the study. The relationship between PNI and renal replacement therapy-free survival (RRTFS) and mortality was examined using logistic regression models, Cox proportional hazard models, and propensity score matching. High utilization rate of parenteral nutrition (PN) was observed in our study. Subgroup analysis was performed to explore the interaction effect between PNI and PN on mortality.

**Results:**

Patients with higher PNI levels exhibited a greater likelihood of achieving RRTFS, with an adjusted odds ratio of 2.43 (95% confidence interval [CI]: 1.98-2.97, *p*-value < 0.001). Additionally, these patients demonstrated higher survival rates, with an adjusted hazard ratio of 0.84 (95% CI: 0.72-0.98) for 28-day mortality and 0.80 (95% CI: 0.69-0.92) for 90-day mortality (all *p*-values < 0.05), compared to those in the low PNI group. While a high utilization rate of parenteral nutrition (PN) was observed, with 78.86% of CRRT patients receiving PN, subgroup analysis showed that high PNI had an independent protective effect on mortality outcomes in AKI patients receiving CRRT, regardless of their PN status.

**Conclusions:**

PNI can serve as an easy, simple, and efficient measure of lymphocytes and albumin levels to predict RRTFS and mortality in AKI patients with require CRRT.

## Introduction

Acute kidney injury (AKI) is characterized by the sudden deterioration of renal function, which results in an increase in serum creatinine levels or a decrease in urine output, according to the definition of the 2012 Kidney Disease Improving Global Outcomes (KDIGO) clinical guideline [1]. AKI is common in patients hospitalized for acute illness, with a prevalence rate of up to 12.8% [2]. Furthermore, AKI affects 52.9%–57.3% of critically ill patients. Reports have highlighted the clinical importance of treating AKI in the intensive care unit (ICU) because it contributes to prolonged ICU stay, increased costs, and a progressively higher mortality rate with higher AKI KDIGO stages [3–5]. Approximately 20%–30% of AKI patients in the ICU require renal replacement therapy (RRT), with continuous RRT (CRRT) being the predominant modality [3,4]. However, the mortality of AKI patients receiving CRRT remains high, ranging between 50% and 70% [6–9].

Nutritional status is a crucial factor for the survival of patients undergoing CRRT. The nutritional requirements of these patients are increased due to the considerable loss of water-soluble electrolytes, glucose, amino acids, and vitamins as well as increases in systemic inflammation, protein catabolism, and heat loss during CRRT [10,11]. Many nutritional factors, including low body mass index (BMI), low initial serum albumin levels, low protein intake, and carnitine deficiency, have been identified to be associated with increased mortality in patients receiving CRRT [12–15].

The prognostic nutritional index (PNI), which combines serum albumin levels and total peripheral blood lymphocyte counts, was originally developed to evaluate the risks of postoperative complications and mortality based on baseline nutritional status in cancer patients undergoing gastrointestinal surgery [16]. The PNI has also been recognized as an indicator of both nutrition and inflammation status [17]. According to our review of the relevant literature, the correlation of the PNI with the mortality rate in patients undergoing CRRT, who represent a unique population with high nutritional demands and increased systemic inflammation, has not been reported previously.

This study evaluated the predictive ability of the PNI for renal replacement therapy–free survival (RRTFS) and 28- and 90-day mortality in patients undergoing CRRT. Additionally, the impact of additional partial parenteral nutrition (PN) supplementation on patient mortality was investigated.

## Materials and methods

### Study populations

This retrospective observational cohort study was conducted at Changhua Christian Hospital (CCH), a tertiary medical center in central Taiwan. This study used data from the Clinical Research Database (CCHRD) of the hospital, which integrates all electronic medical records pertaining to the CRRT database, prescriptions, laboratory results, clinical visit records, and death records. This study analyzed the data of 3033 intensive care unit (ICU) patients who received CRRT between January 1, 2010, and April 30, 2021.

**Figure 1.**
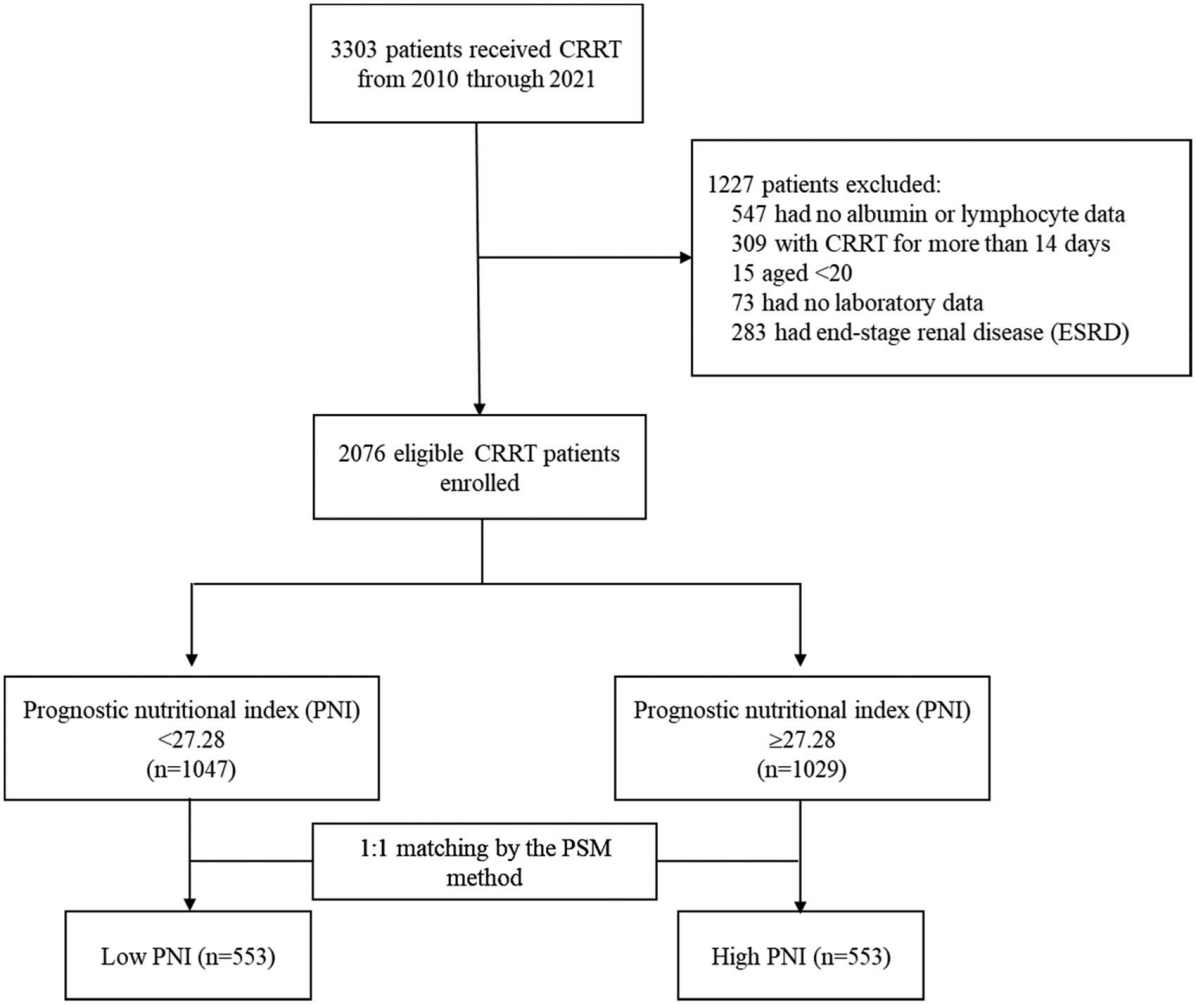
Flow chart.

To accurately assess the role of nutritional status in the clinical outcomes of patients undergoing CRRT in the ICU, we excluded patients who had incomplete biochemical data, were younger than 20 years, and had end-stage renal disease. Renal failure requiring prolonged CRRT may involve additional complicating factors compared with typical renal failure. These factors include disease severity, a complex medical history, and the need for more intensive medical care and monitoring [18,19]. These complex factors affect nutritional status and clinical outcomes, and controlling for them during analysis is difficult [19–22]. Therefore, patients who received prolonged CRRT were excluded from this study, and this study focused on investigating the association between nutrition status and clinical outcomes among patients with similar treatment courses. Ultimately, a total of 2076 patients were included in the analysis (Figure 1). The Institutional Review Board of CCH granted a waiver for the informed consent requirement and approved this study (IRB No: 220819).

### Nutritional measurements and other confounders

The main focus of this study was the patient’s nutritional status on CRRT initiation. Nutritional status was measured using the PNI, which was developed by Onodera et al. [16], and the PNI was calculated using the following formula:

$$10\;\times \text{ serum albumin }\left(g/\text{dL}\right)\;+\;0.005\;\times \text{ total lymphocyte count }\left(/{\text{mm}}^{3}\right).$$

The study population was divided into two distinct groups based on the established median values of the PNI: low PNI (<27.28) and high PNI (≥27.28).

The demographic and clinical information of patients, including age, sex, BMI, Acute Physiology and Chronic Health Evaluation (APACHE) II score at ICU admission, diagnostic criteria for AKI, the timing of CRRT initiation, vital signs within 24 h of CRRT initiation, urine output, pre-CRRT medication, serum biochemical data, presence of comorbidities, and use of PN, was collected from the CCHRD. These clinical confounders have been discussed in previous articles [9, 23].

### Endpoint

The primary endpoint in this study was RRTFS, which was defined as patients who were discharged alive after undergoing CRRT and who did not require RRT for 7 or more consecutive days before discharge. The secondary endpoint was the mortality rate at 72 h, 28 days, and 90 days of receiving CRRT as well as the renal outcome within 365 days. The index date was defined as the date of CRRT initiation. Patient data were censored at the date of discharge, 28 days and 90 days of receiving CRRT, time of initiation of palliative care, or the last available follow-up date, whichever occurred first.

### Statistical analysis

Categorical variables are expressed as numbers (proportions), and continuous variables are expressed as medians and interquartile ranges (IQRs). Categorical variables were compared using the chi-square test, and continuous variables were compared using the Mann–Whitney U test. To account for confounders in the PNI group, a multivariate logistic regression model with backward elimination was used to calculate the propensity score for each patient based on their demographic and characteristic covariates. A 1:1 propensity score–matched analysis was conducted using the nearest-neighbor method with a caliper of 0.1 SD units to create matched pairs. The standardized mean difference (SMD) was used to examine the balance in the distribution of covariates between the two PNI groups, with an SMD of >0.1 indicating imbalance. The association of the RRTFS of the PNI groups was assessed using crude and multivariate logistic regression models. The mortality rates during the follow-up period according to the PNI groups were estimated using crude and multivariate Cox proportional hazard models. Regression models were applied both before and after propensity score matching to enhance the reliability of the results. Restricted cubic splines were used to evaluate the association of PNI levels with RRTFS and mortality. The survival curve was analyzed nonparametrically by using the Kaplan–Meier method, and the analysis was stratified by PNI categories; differences were evaluated using log-rank tests. In our study, a high utilization rate of parenteral nutrition (PN) was observed, with 78.86% of CRRT patients receiving PN. Furthermore, we investigated the relationship between PNI and mortality, considering potential interactions with parenteral nutrition (PN) status. Subgroup analyses were conducted to assess the impact of PNI on mortality among patients receiving and those not receiving PN. Forest plot visualized the result of subgroup analysis. To enhance the robustness of the findings and to achieve a comprehensive understanding of the relationship between PNI and clinical outcomes, we conducted a series of sensitivity analyzes based on various scenarios. Receiver operating characteristic (ROC) analysis was performed and the area under the curve (AUC) was calculated to compare the predictive performance of PNI with albumin and lymphocytes. Nutritional indicators were standardized and transformed to eliminate unit variation. The relevant analysis is attached in the supplementary information section. All statistical analyses and descriptions were performed using SAS, and the visualization plot was created using R software (version 4.1.0; The Comprehensive R Archive Network: http://cran.r-project.org). A two-sided *p* value of <0.05 was considered statistically significant.

## Results

### Baseline characteristics of study cohort

This study included a total of 2076 patients who underwent CRRT. The median PNI was 27.28 (IQR, 21.52–33.47). Among the included patients, 550 (26.5%) achieved RRTFS. The baseline characteristics of patients, stratified by the PNI groups, are presented in Table 1.

**Table 1.** Demographic.

	Before propensity score matching			After propensity score matching			
	High PNI	Low PNI	*P*-value	High PNI	Low PNI	*P*-value	SMD
Sample size	1029	1047		553	553		
Gender, Male	668 (64.9%)	675 (64.5%)	0.831	358 (64.7%)	351 (63.5%)	0.699	0.026
Age	69 (58-80)	71 (60-81)	0.029	72 (61.5-82)	71 (61-81)	0.515	0.042
BMI, kg/m^2^	25.0 (21.9-28.6)	23.4 (20.6-26.9)	<0.001	24.3 (21.3-28.1)	24.6 (21.6-28.0)	0.974	0.007
APACHE II at admission	29 (22-35)	29 (23-36)	<0.001	29 (22-35)	29 (23-34)	0.521	0.017
Diagnostic criteria for AKI							
Oliguria	293 (28.47%)	335 (32%)	0.081	168 (30.4%)	162 (29.3%)	0.752	0.024
Anuria	242 (23.52%)	217 (20.73%)	0.125	127 (23.0%)	118 (21.3%)	0.571	0.041
AKI achieves KDIGO-defined serum Creatine elevation	247 (24%)	318 (30.37%)	0.001	152 (27.5%)	164 (29.7%)	0.474	0.049
Others	247 (24%)	177 (16.91%)	<0.001	106 (19.2%)	109 (19.7%)	0.878	0.013
Timing of initiated CRRT							
Early strategy	622 (60.45%)	491 (46.9%)	<0.001	279 (50.5%)	286 (51.7%)	0.721	0.025
Delayed strategy	407 (39.55%)	556(53.1%)		274 (49.5%)	267 (48.3%)		
Vital sign							
Systolic BP (mmHg)	111.8 (101.1-123.9)	108.1(99.7-119.4)	<0.001	110.0 (100.7-120.2)	109.8 (100.7-122.3)	0.147	0.105
Diastolic BP (mmHg)	58.8 (51.4-68.1)	56.8 (50.2-63.9)	<0.001	56.6 (49.3-65.1)	57.8 (51.7-64.0)	0.911	0.024
Pulse rate (bpm)	101.4 (85.1-115.8)	105.0 (90.5-117.8)	<0.001	102.4 (87.1-117.3)	105.3 (90.0-118.0)	0.145	0.089
Body temperature (degree Celsius)	36.2 (35.4-36.9)	36.2 (35.4-36.8)	0.662	36.4 (35.7-37.0)	36.3 (35.6-36.9)	0.272	0.036
Respiratory rate (/min)	19.7 (16.8-22.5)	20.3 (17.3-23.8)	<0.001	20.3 (17.2-23.0)	20 (17.1-23.1)	0.795	0.013
SPO_2_	97.3 (94.6-98.9)	96.6 (93.7-98.5)	<0.001	96.8 (94.4-98.5)	96.8 (94.1-98.4)	0.544	<0.001
Multiple organ support before CRRT-no. (%)							
Invasive mechanical ventilation	866 (84.2%)	937 (89.5%)	<0.001	479 (86.6%)	478 (86.4%)	1.000	0.005
Extracorporeal Membrane Oxygenation (ECMO)	132 (12.8%)	58 (5.5%)	<0.001	45 (8.1%)	39 (7.1%)	0.561	0.041
Vasopressors support with norepinephrine or epinephrine	815 (79.2%)	944 (90.2%)	<0.001	484 (87.5%)	480 (86.8%)	0.789	0.022
Medication use before CRRT-no. (%)							
Sedative	736 (74.5%)	780 (71.5%)	0.127	396 (71.6%)	390 (70.5%)	0.736	0.024
Corticosteroids	539 (52.4%)	721 (68.9%)	<0.001	333 (60.2%)	343 (62.0%)	0.548	0.037
Loop diuretic	1047 (100%)	1029 (100%)	--	553 (100%)	553 (100%)	--	<0.001
Furosemide	551 (53.6%)	621 (59.3%)	0.008	317 (57.3%)	295 (53.3%)	0.200	0.080
Antibiotics	957 (93%)	1023 (97.7%)	<0.001	540 (97.6%)	537 (97.1%)	0.710	0.034
Urine Output before CRRT—ml/24 hours	14.8 (3.8-39.6)	12.9 (4.0-33.3)	0.153	15.8 (3.8-41.4)	12.3 (3.3-31.3)	0.140	0.151
Fluid balance before CRRT—ml/24 hours	1836.1 (728.5-3194)	2249.5 (1040-3957)	<0.001	2199 (1074-3637)	2083 (1060-3750)	0.699	0.005
Coexisting conditions-no. (%)							
Hypertension	392 (38.1%)	398 (38.0%)	0.969	215 (38.9%)	210 (38.0%)	0.804	0.019
Diabetes Mellitus	379 (36.8%)	369 (35.2%)	0.451	209 (37.8%)	200 (36.2%)	0.611	0.034
Hyperlipidemia	205 (19.9%)	161 (15.4%)	0.007	98 (17.7%)	97 (17.5%)	1.000	0.005
Coronary artery disease	291 (28.3%)	183 (17.5%)	<0.001	118 (21.3%)	109 (19.7%)	0.518	0.040
Congestive heart failure	216 (21.0%)	145 (13.9%)	<0.001	98 (17.7%)	97 (17.5%)	1.000	0.005
Chronic pulmonary disease	197 (19.1%)	198 (18.92%)	0.892	120 (21.7%)	105 (19.0%)	0.290	0.067
Chronic renal disease	377 (36.6%)	351 (33.5%)	0.137	205 (37.1%)	204 (36.9%)	1.000	0.004
Malignancy	20 (1.9%)	46 (4.4%)	0.001	15 (2.7%)	16 (2.9%)	1.000	0.011
Leukemia	17(1.7%)	17(1.6%)	0.959	12(2.2%)	6(1.1%)	0.154	0.086
Lymphoma	12(1.2%)	27(2.6%)	0.018	6(1.1%)	18(3.3%)	0.013	0.149
Chronic Liver disease	182(17.7%)	206(19.7%)		114(20.6%)	111(20.1%)	0.823	0.012
Cardiac arrhythmia occurrence at the baseline	270 (26.2%)	200 (19.1%)	<0.001	118 (21.3%)	117 (21.2%)	1.000	0.004
Cardiac arrhythmia occurrence during the 24-hour CRRT	144 (14.0%)	81 (7.7%)	<0.001	50 (9.0%)	57 (10.3%)	0.534	0.043
Laboratory data before CRRT							
Albumin, g/dL	2.8 (2.5-3.2)	1.9 (1.5-2.2)	<0.001	2.8 (2.6-3.1)	1.9 (1.6-2.2)	<0.001	1.876
Hemoglobin, g/dL	10.2 (8.6-12.1)	9.4 (8.3-10.6)	<0.001	9.6 (8.4-11.1)	9.6 (8.4-10.9)	0.768	0.031
WBC count, 1000/μL	12.2 (8.6-17.5)	10.5 (6.0-16.6)	<0.001	11.6 (8.4-17.0)	11.8 (7.1-19.5)	0.860	0.013
Lymphocyte count, 1000/μL	0.96 (0.50-1.86)	0.39 (0.19-0.72)	<0.001	0.96 (0.47-1.77)	0.43 (0.22-0.75)	<0.001	0.744
Platelet count, 1000/μL	128 (74-201)	86 (44-158)	<0.001	107 (60-176.5)	107 (53-184)	0.688	0.007
pH	7.3 (7.2-7.4)	7.3 (7.2-7.4)	0.139	7.3 (7.2-7.4)	7.3 (7.2-7.4)	0.630	0.052
Sodium, mmol/L	138 (134-142.4)	138 (134-143)	0.665	138 (135-143)	138 (134-143)	0.246	0.067
Lactate, mmol/L	4.1 (1.8-9.4)	4.1 (2.0-8.7)	0.994	3.2 (1.6-8.3)	3.7 (1.8-7.3)	0.761	0.040
K, mmol/L	4.0 (3.5-4.7)	3.9 (3.4-4.7)	0.266	4.0 (3.4-4.7)	3.9 (3.5-4.8)	0.201	0.053
Calcium, mg/Dl	8.0 (7.4-8.6)	7.6 (7.0-8.2)	<0.001	7.9 (7.4-8.5)	7.8 (7.3-8.4)	0.411	0.018
Base Excess, mmol/L	−7.3 (-11.3- −4.2)	−8.1 (-11.7- −4.9)	0.006	−7.0 (-10.9--4.0)	−7.9 (-11.1--4.5)	0.178	0.073
O_2_ Saturation, %	98.4 (95.4-99.7)	98 (95.5-99.5)	0.026	98.4 (95.9-99.6)	97.8 (95.6-99.3)	0.699	0.009
Creatinine, mg/dL	2.1 (1.3-4.3)	2.2 (1.3-3.8)	0.304	1.9 (1.2-3.9)	2.2 (1.3-3.7)	0.585	0.019
Nutritional supplement							
Total parenteral nutrition	84 (8.16%)	156 (14.9%)	<0.001	61 (11.0%)	61 (11.0%)	1.000	0
Parenteral nutrition	803 (78.04%)	790 (75.45%)	0.164	437 (79.0%)	435 (78.7%)	0.942	0.007
None	142 (13.8%)	101 (9.65%)	0.003	55 (9.9%)	57 (10.3%)	0.920	0.013
Modality of CRRT							
CVVH	957(93.0%)	916(87.5%)	<0.001	508(91.9%)	495(89.5%)	0.179	0.081
CVVHD	72(7.0%)	131(12.5%)		45(8.1%)	58(10.5%)		
Outcome							
RRTFS	360 (35.0%)	190 (18.1%)	<0.001	172 (31.1%)	120 (21.7%)	1.000	0.215
72-hour mortality	348 (33.8%)	441 (42.1%)	<0.001	212 (38.3%)	219 (39.6%)	0.717	0.026
28-day mortality	562 (54.6%)	764 (73.0%)	<0.001	334 (60.4%)	389 (70.3%)	0.001	0.210
90-day mortality	617 (60.0%)	836 (79.9%)	<0.001	365 (66.0%)	426 (77.0%)	<0.001	0.246

SMD: standardized mean difference used to examine the balance in the distribution of covariates between the two PNI groups, with an SMD of >0.1 indicating an imbalance.

Prior to conducting propensity score matching, many demographic and clinical characteristics were statistically significantly different between the PNI groups. These characteristics included age, BMI, APACHE II score at admission, the timing of CRRT initiation, vital signs at CRRT initiation, multiple organ support before CRRT, medication use, coexisting conditions, the occurrence of cardiac arrhythmia during the 24-h CRRT, and laboratory data before CRRT. Of the 2076 patients, 1047 had a low PNI, whereas 1029 had a high PNI. The 1:1 propensity score matching generated 553 matched pairs for the low and high PNI groups (Figure 1). The variables did not exhibit statistically significant differences between the PNI groups, except for albumin.

### Renal replacement therapy–free survival

**Figure 2.**
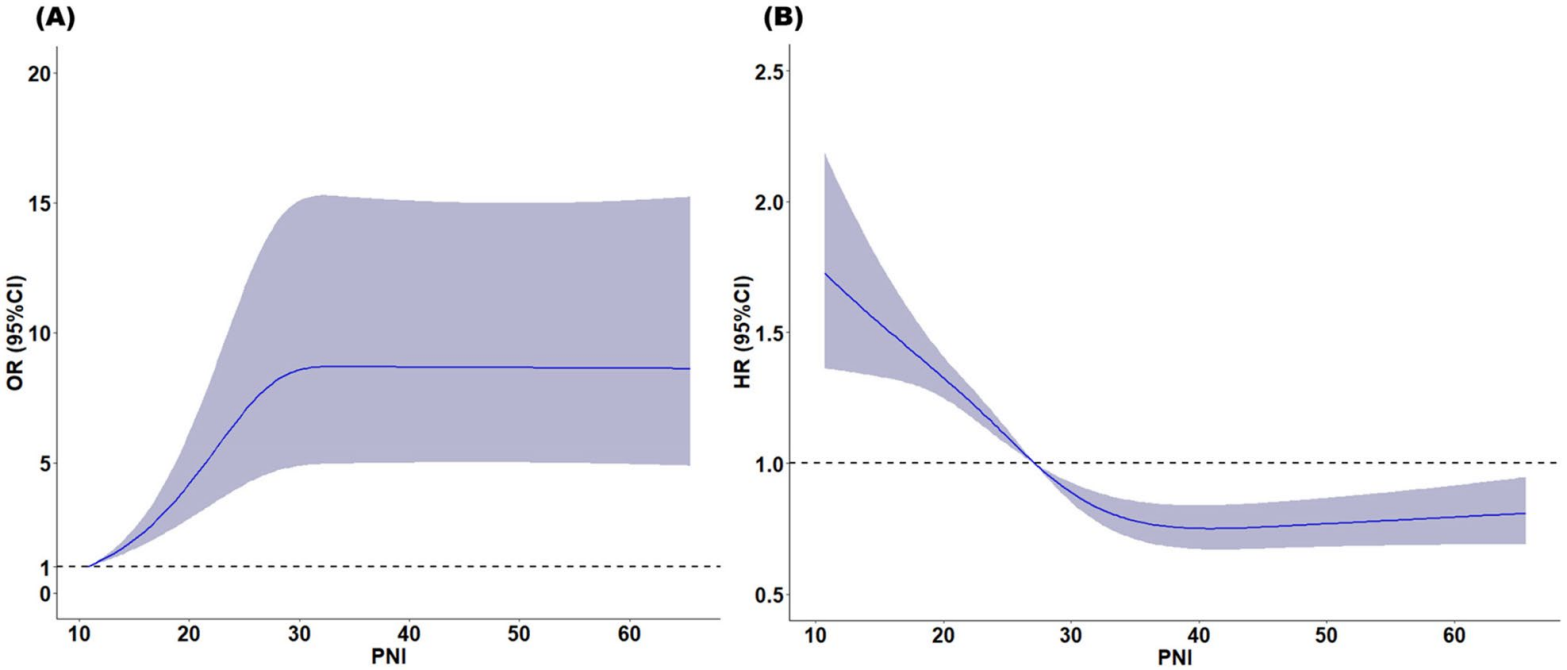
Restricted cubic spline analysis to investigate the relationship between PNI and (A) RRTFS (B) 90-day mortality.

We observed a positive and significant association between PNI levels (as a continuous variable) and RRT-free survival according to restricted cubic splines (Figure 2a). The results of the univariate and multivariate logistic regression analyses revealed that patients in the high PNI group had a significantly increased likelihood of RRTFS compared with those in the low PNI group (Table 2). In the univariate analysis, the crude odds ratio (OR) for RRTFS in the high PNI group was 2.43 (95% confidence interval [CI] = 1.98–2.97; *p* < 0.001). After various covariates were adjusted for in the multivariate analysis, the adjusted OR for a high PNI was 1.76 (95% CI = 1.29–2.40; *p* < 0.001). Similar results were observed in the propensity score matching analysis (Table 2).

**Table 2.** Odds ratio (or) and 95% confidence interval (CI) for RRTFS according to PNI.

	RRTFS			
	cOR (95%CI)	*P*-value	aOR (95%CI)	*P*-value
***Before propensity score matching (n = 2076)***				
PNI as continuous				
PNI per 1-unit increase	1.02(1.01,1.03)	<0.001	1.03(1.02,1.05)	<0.001
Dichotomy of PNI				
Low PNI (PNI < 27.28)	1 (reference group)		1 (reference group)	
High PNI (PNI ≥ 27.28)	2.43 (1.98,2.97)	<0.001	1.76 (1.29,2.40)	<0.001
***After Propensity Score Matching (n = 1066)***				
PNI as continuous				
PNI per 1-unit increase	1.02(1,1.03)	0.016	1.01(1,1.03)	0.041
Dichotomy of PNI				
Low PNI (PNI < 27.28)	1 (reference group)		1 (reference group)	
High PNI (PNI ≥ 27.28)	1.63 (1.24,2.14)	<0.001	1.64 (1.25,2.14)	<0.001

OR, odds ratio; CI, confidence interval; cOR, crude odds ratio; aOR, the multivariate-adjusted odds ratio, the variables with a *p*-value < 0.05 in a univariate model were included in the multivariate model.

### Seventy-two-hour, 28-day, 90-day mortality and 365-day renal outcome after receiving CRRT

**Figure 3.**
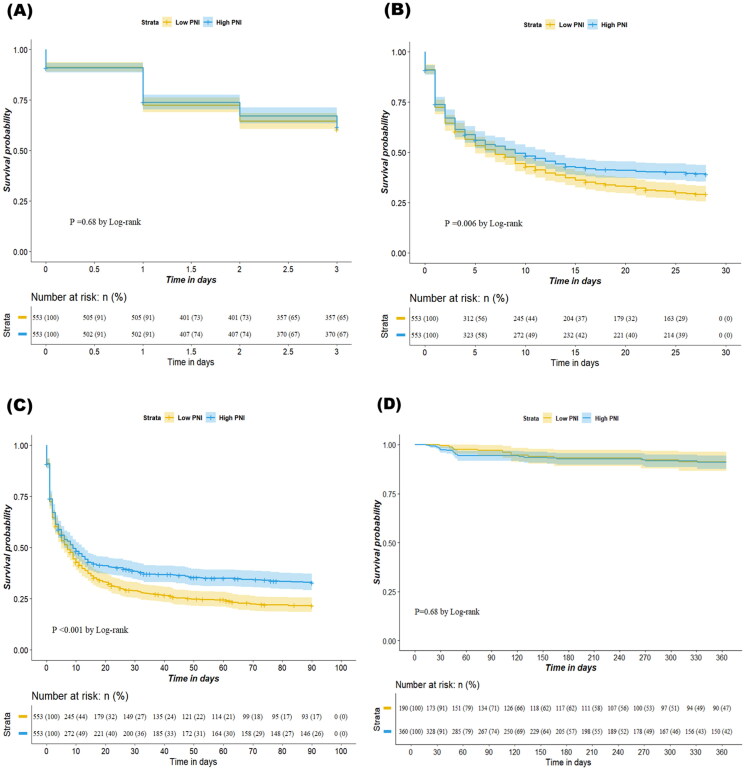
Kaplan-Meier Curves for (A) 72 h mortality (B) 28 days mortality, (C) 90 days mortality and (D) renal outcome using propensity score matching dataset.

We observed a negative and significant association between PNI levels (as a continuous variable) and 90-day mortality according to restricted cubic splines (Figure 2b). The results of the Kaplan–Meier analysis revealed that a higher PNI was associated with improved 28-day and 90-day survival rates compared with a lower PNI (*p* = 0.006 for 28-day mortality; *p* < 0.001 for 90-day mortality; Figure 3b,c). Cox proportional hazard models were used to assess the mortality risk associated with the PNI. In the unadjusted model, the high PNI group exhibited lower 28-day and 90-day mortality risks (hazard ratio [HR] = 0.65, 95% CI = 0.59–0.73, *p* < 0.001 for 28-day mortality; HR = 0.63, 95% CI = 0.57–0.70, *p* < 0.001 for 90-day mortality). After adjustment of various covariates in the multivariate model, the association between a high PNI and reduced mortality risk remained significant (adjusted hazard ratio [aHR] = 0.84, 95% CI = 0.72–0.98, *p* = 0.029 for 28-day mortality; aHR = 0.80, 95% CI = 0.69–0.92, *p* = 0.002 for 90-day mortality). Similar results were obtained in the propensity score matching model (aHR = 0.81, 95% CI = 0.70–0.94, *p* = 0.005 for 28-day mortality; aHR = 0.79, 95% CI = 0.68–0.90, *p* < 0.001 for 90-day mortality; Table 3). However, the 72-h survival rates and 365-day renal outcome did not significantly differ between the high and low PNI groups (Figure 3a,d). Although a higher PNI was significantly correlated with a lower 72-h mortality rate in the unadjusted model (HR = 0.77, 95% CI = 0.67–0.88, *p* < 0.001), the association was nonsignificant in the multivariate model (aHR = 1.00, 95% CI = 0.82–1.21, *p* = 0.975) or the propensity score–matching model (aHR = 0.98, 95% CI = 0.79–1.16, *p* = 0.668; Table 3). Higher PNI was not significantly associated with renal outcomes in either the unadjusted model (HR = 1.02, 95% CI = 0.53–1.97, *p* = 0.953) or the multivariable model (aHR = 0.98, 95% CI = 0.48–2.03, *p* = 0.961; Supplementary Table S3).

**Table 3.** Hazard ratio (HR) and 95% confidence interval (CI) for 72-h mortality/28-day mortality/90-day mortality according to PNI.

	72-hr mortality				28-day mortality				90-day mortality			
	cHR (95%CI)	*P*-value	aHR (95%CI)	*P*-value	cHR (95%CI)	*P*-value	aHR (95%CI)	*P*-value	cHR (95%CI)	*P*-value	aHR (95%CI)	*P*-value
***Before propensity score matching (Overall)***												
PNI as continuous	0.99 (0.99,1.00)	0.049	1.00 (0.99,1.01)	0.810	0.99 (0.98,0.99)	<0.001	0.99 (0.98,1)	0.003	0.98 (0.98,0.99)	<0.001	0.99 (0.98,0.99)	<0.001
Dichotomy of PNI												
Low PNI	1		1		1		1		1		1	
High PNI	0.77 (0.67,0.88)	<0.001	1.00 (0.82,1.21)	0.975	0.65 (0.59,0.73)	<0.001	0.84 (0.72,0.98)	0.029	0.63 (0.57,0.70)	<0.001	0.80 (0.69,0.92)	0.002
***After propensity score matching***												
PNI as continuous	1(0.99,1.01)	0.843	1(0.99,1.01)	0.753	0.99(0.98,1)	0.053	0.99(0.99,1)	0.180	0.99(0.98,1)	0.011	0.99(0.98,1)	0.046
Dichotomy of PNI												
Low PNI	1		1		1		1		1		1	
High PNI	0.96 (0.80,1.16)	0.701	0.96 (0.79,1.16)	0.668	0.82 (0.71,0.95)	0.009	0.81 (0.70,0.94)	0.005	0.80 (0.69,0.92)	0.002	0.79 (0.68,0.90)	<0.001

HR, hazard ratio; CI, confidence interval; cHR, crude hazard ratio; aHR, the multivariate-adjusted hazard ratio, the variables with a *p*-value < 0.05 in a univariate model were included in the multivariate model.

### Subgroup analysis

**Figure 4.**
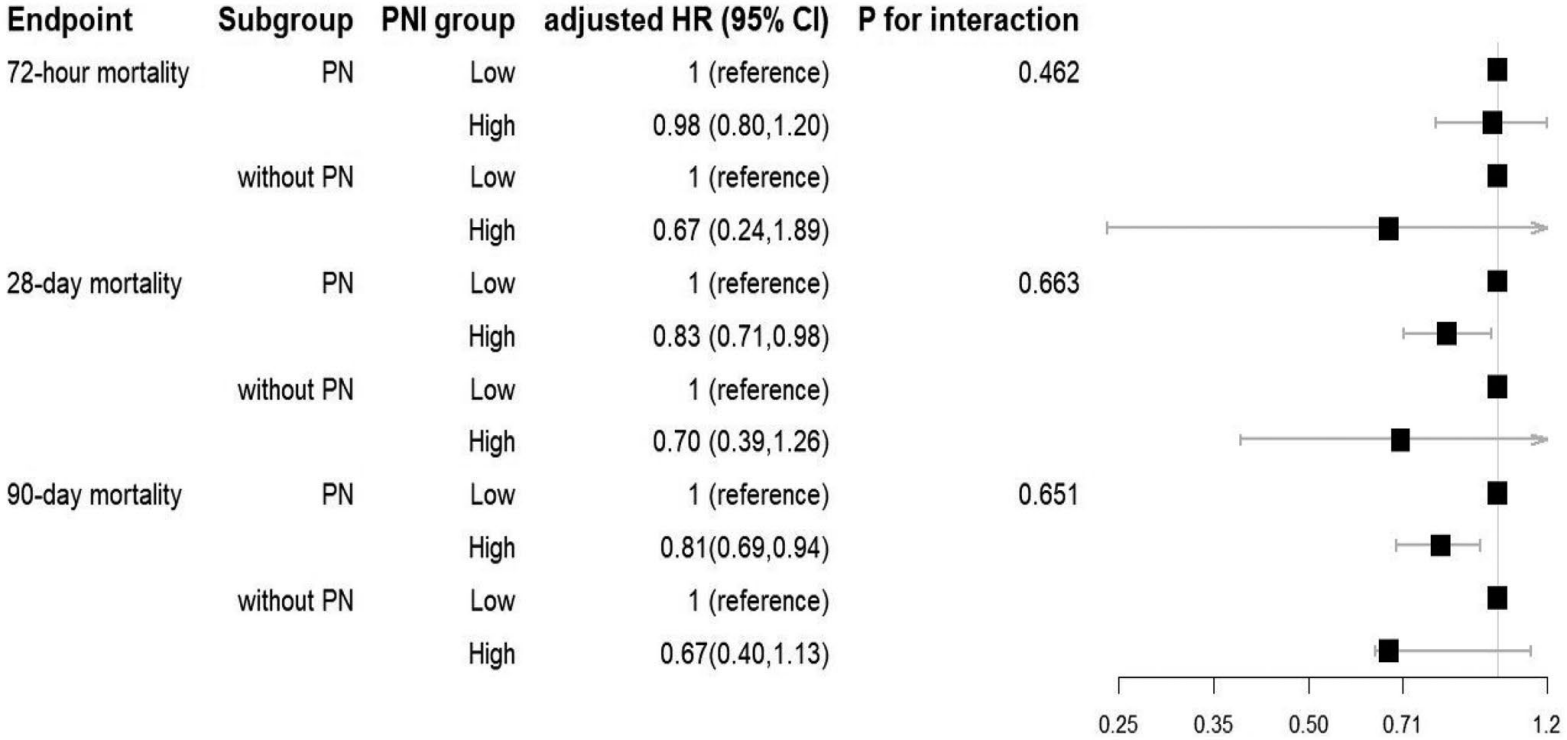
Forest Plot illustrating subgroup analysis results.

While a high utilization rate of parenteral nutrition (PN) was observed, with 78.86% of CRRT patients receiving PN, subgroup analysis was performed to explore whether there is an interaction between PNI and PN on mortality. Our analysis revealed that high PNI patients who received parenteral nutrition exhibited significantly lower hazard ratios for mortality compared to low PNI patients who received PN (Figure 4.). However, we found no significant difference in the association between PNI and mortality among patients with and without who received PN, as indicated by the non-significant *p*-value for the interaction term between PNI level and PN status. These results suggest that high PNI may confer a protective effect on mortality outcomes, regardless of PN status.

### Sensitivity analysis

To enhance the robustness of the findings, we conducted a series of sensitivity analyzes based on various scenarios (Table 4). First, all patients, regardless of CRRT duration, were included. Supplementary Table S1 shows substantial differences in patient clinical characteristics and mortality between CRRT duration groups. Sensitivity analysis revealing a significant association between high PNI and increased RRTFS odds ratio, with an adjusted OR of 1.76 (95% CI: 1.29, 2.40). Additionally, high PNI was associated with reduced 28-day and 90-day mortality, with an adjusted HRs of 0.84 (95% CI: 0.72, 0.98) and 0.80 (95% CI: 0.69, 0.92), respectively. Secondly, we employed Youden’s index to categorize high and low levels. In our dataset, the cutoff value derived from the Youden index was found to be 27.34, closely resembling our original cutoff value of 27.28 determined *via* the median. Furthermore, this multivariate-adjusted analysis yielded consistent results with our primary analysis. Thirdly, PNI was divided into quartiles. Multivariable adjusted analysis revealed that while the second quartile showed no significant association with clinical outcomes, the third and fourth quartiles remained significantly associated with these outcomes.

**Table 4.** Sensitivity analysis.

	RRTFS outcome		72-hr mortality		28-day mortality		90-day mortality	
	Adjusted OR (95%CI)	*P*-value	Adjusted HR (95%CI)	*P*-value	Adjusted HR (95%CI)	*P*-value	Adjusted HR (95%CI)	*P*-value
Included patients with CRRT more than 14 days (*N* = 2385)								
Low PNI (PNI < 27.28)	1(reference)		1(reference)		1(reference)		1(reference)	
High PNI (PNI ≥ 27.28)	1.76 (1.29,2.40)	<0.001	1.00 (0.82,1.21)	0.975	0.84 (0.72,0.98)	0.030	0.80 (0.69,0.92)	0.002
Youden’s index cutpoint								
Low PNI (PNI < 27.34)	1(reference)		1(reference)		1(reference)		1(reference)	
High PNI (PNI ≥ 27.34)	1.76 (1.29,2.40)	<0.001	1.00 (0.82,1.21)	0.975	0.84 (0.72,0.98)	0.030	0.80 (0.69,0.92)	0.002
Quartile of PNI								
PNI < 21.5	1(reference)		1(reference)		1(reference)		1(reference)	
21.5 to <27.28	1.85 (1.17,2.92)	0.008	0.98 (0.75,1.28)	0.876	1.01 (0.84,1.21)	0.933	0.95 (0.79,1.14)	0.576
27.28 to <33.5	2.51 (1.60,3.96)	<0.001	1.08 (0.84,1.38)	0.562	0.82 (0.67,1.00)	0.048	0.80 (0.66,0.97)	0.022
≥33.5	2.63 (1.64,4.23)	<0.001	1.13 (0.86,1.48)	0.398	0.81 (0.65,1.00)	0.048	0.74 (0.61,0.92)	0.005

## Discussion

This study evaluated the predictive ability of PNI for RRTFS and 72-h, 28-day, and 90-day mortality, and. Our findings revealed that the high PNI group had a significantly higher crude OR and adjusted OR for RRTFS than the low PNI group. These results suggest that patients with higher PNI levels were more likely to achieve RRT-free survival. Furthermore, our results revealed a significant association between PNI and 28- and 90-day mortality in patients undergoing CRRT. The high PNI group exhibited a lower HR than the low PNI group, suggesting that patients with better nutritional and inflammatory status had improved survival rates. These findings are consistent with those of studies that have identified low serum albumin levels and low protein intake as independent predictors of mortality in critically ill patients [13,14].

The PNI has been demonstrated to be a useful predictor of various outcomes, including survival, complications, and response to treatment, in patients with chronic disease and cancer. Moreover, lower PNI has been shown to be significantly associated with higher mortality in patients with cancer [24–26], cardiovascular disease [27,28], or neonatal sepsis [29]. Haneda et al. [30] demonstrated that the PNI was an independent predictor of mortality in patients with esophageal cancer undergoing esophagectomy. Similarly, Wang et al. [31] revealed that the PNI was a significant independent predictor of postoperative complications in patients with head and neck cancer undergoing surgical resection.

Our study extends these findings to the unique population of patients undergoing CRRT, who have both a high nutritional demand and increased systemic inflammation.

Albumin is commonly employed to evaluate malnutrition, particularly in chronic kidney disease patients undergoing dialysis. Prior studies have highlighted the importance of the PNI across various diseases. For instance, Dolapoglu et al. [32] and Hu et al. [28] observed a significant correlation between PNI and AKI in patients undergoing coronary procedures. However, the potential utility of PNI in AKI patients undergoing RRT remains unexplored. In our study, we aimed to fill this gap by evaluating the role of PNI as a nutritional index in patients with AKI on CRRT. While ROC analysis suggested albumin’s superior discriminatory ability (Supplementary Figure S1), PNI also showed good predictive value in our cohort. Notably, standardized PNI was associated with higher RRTFS likelihood and lower hazard ratio of death (Supplementary Table S2). These findings indicate that, despite potential differences in ROC performance, PNI provides valuable prognostic insights for AKI patients undergoing CRRT.

We found that the median cutoff value of PNI was 27.28, which is consistent with the results of previous studies, which reported a PNI of 28 for critically ill patients with COVID-19 [33] and the mean PNI value of 26.6 (21.26–33.72) for patients with septic AKI [34]. Notably, critically ill patients had lower PNI cutoff values than noncritically ill patients. Although the mechanism underlying the association between the PNI and mortality risk is not fully understood, it may involve the interplay between nutrition and inflammation. Malnutrition can lead to immunosuppression and impaired wound healing, which can increase the risks of infection and other complications [35,36]. In addition, malnutrition can trigger an inflammatory response, which can lead to tissue damage and organ dysfunction. The PNI, as a composite index encompassing both nutritional status and immune function, may provide a more comprehensive assessment of a patient’s overall health status and disease severity.

Nutritional support, particularly parenteral nutrition (PN), is vital in managing acute kidney injury (AKI), especially in patients undergoing continuous renal replacement therapy (CRRT). Current guidelines emphasize the importance of initiation of parenteral nutrition when enteral nutrition cannot meet energy and protein requirements, aiming to prevent protein-energy wastage and its associated complications [37–39]. Although the benefits of nutritional support are well recognized, a personalized and carefully integrated approach is crucial [39]. In our study, 78.86% of CRRT patients received PN, highlighting its prevalence. To explore whether there is an interaction between PNI and PN on mortality, we performed a subgroup analysis. Our findings suggest that high PNI independently exerts a protective effect on mortality outcomes in AKI patients receiving CRRT, regardless of their PN status. Although PN is established to meet nutritional needs, our results indicate that the beneficial effects of high PNI on mortality persist regardless of PN support.

This study has several limitations that should be considered when interpreting the results. First, an extensive electronic database from a single center was used in this study, which may limit the generalizability of the results to other patient populations. Second, because this was a retrospective observational study, the causal relationship of the PNI with RRTFS and mortality could not be determined. Third, although the study used propensity score matching to adjust for some possible confounders, some residual confounders may not have been considered. Fourth, our study lacked identification of the etiology of acute kidney injury (AKI). While the cause of AKI is a crucial factor influencing outcomes, it is often multifactorial, and precise identification can be challenging, especially in critically ill patients within the ICU setting. Fifth, our study lacked repeated PNI values over time to analyze changes in PNI before and after CRRT initiation. Lastly, because this study included ICU patients with different causes of AKI, their albumin and lymphocyte levels may differ depending on individual conditions, and additional large-scale studies are required to validate the results.

## Conclusion

In conclusion, patients with higher PNI levels exhibited greater likelihood of achieving RRTFS and higher survival rates compared to those in the low PNI group. While a high utilization rate of parenteral nutrition (PN) was observed, with 78.86% of CRRT patients receiving PN, subgroup analysis showed that high PNI had an independent protective effect on mortality outcomes in AKI patients receiving CRRT, regardless of their PN status. Our findings suggest that the PNI can serve as an easy, simple, and efficient measure of lymphocytes and albumin levels to predict RRTFS and mortality in AKI patients with require CRRT. Clinicians may consider integrating PNI assessment into routine prognostic evaluations to identify high-risk patients and tailor therapeutic strategies accordingly.

## Supplementary Material

Supplemental Material

## Data Availability

The data that support the findings of this study originate from Changhua Christian Hospital clinical research database. Restrictions apply to the availability of these data and they are therefore not publicly available.
