# Supplementary material for: Impact of the prognostic nutritional index on renal replacement therapy–free survival and mortality in patients on continuous renal replacement therapy
Source: Ren Fail. 2024 Jun 14;46(2):2365394. doi: 10.1080/0886022X.2024.2365394 (PMC11232640; doi:10.1080/0886022X.2024.2365394)
Supplement: Supplemental Material [file IRNF_A_2365394_SM4065.docx]

**Supplementary Material**

Table S1. Demographic and clinical variables between patients receiving CRRT within 14 days and more than 15 days.

|  | **CRRT duration ≤ 14 days** | **CRRT duration ≥15 days** | **P-value** |
| --- | --- | --- | --- |
| **Sample size** | **2076** | **309** |  |
| Gender, Male | 1343(64.7%) | 201(65%) | 0.903 |
| Age | 70(59,81) | 66(58,77) | 0.010 |
| BMI, kg/m^2^ | 24.2(21.3,27.6) | 25.4(22,28.7) | 0.003 |
| APACHE II at admission | 29(22,36) | 28(22,34) | 0.071 |
| Diagnostic criteria for AKI |  |  | <0.001 |
| Oliguria | 628(30.3%) | 116(37.5%) |  |
| Anuria | 459(22.1%) | 69(22.3%) |  |
| AKI achieves KDIGO-defined serum Creatine elevation | 565(27.2%) | 91(29.5%) |  |
| Others | 424(20.4%) | 33(10.7%) |  |
| Timing of initiated CRRT |  |  |  |
| Early strategy | 1113(53.6%) | 119(38.5%) | <0.001 |
| Delayed strategy | 963(46.4%) | 190(61.5%) |  |
| Vital sign |  |  |  |
| Systolic BP (mmHg) | 109.6(100.4,121.1) | 115(104.4,127.7) | <0.001 |
| Diastolic BP (mmHg) | 57.9(50.7,65.8) | 60.4(53.5,68.8) | <0.001 |
| Pulse rate (bpm) | 103.3(87.7,116.8) | 99.9(85.4,113.8) | 0.017 |
| Body temperature (degree Celsius) | 36.2(35.4,36.9) | 36.3(35.7,36.9) | 0.073 |
| Respiratory rate (/min) | 20(16.9,23.1) | 18.2(15.7,21) | <0.001 |
| SPO_2_ | 96.9(94.2,98.7) | 97.5(95.8,99.1) | <0.001 |
| Multiple organ support before CRRT-no. (%) |  |  |  |
| Invasive mechanical ventilation | 1803(86.8%) | 288(93.2%) | 0.002 |
| Extracorporeal Membrane Oxygenation (ECMO) | 190(9.2%) | 58(18.8%) | <0.001 |
| Vasopressors support with norepinephrine or epinephrine | 1759(84.7%) | 250(80.9%) | 0.085 |
| Medication use before CRRT-no. (%) |  |  |  |
| Sedative | 1516(73%) | 265(85.8%) | <0.001 |
| Corticosteroids | 1260(60.7%) | 205(66.3%) | 0.057 |
| Loop diuretic | 2076(100%) | 309(100%) | 1.000 |
| Furosemide | 1172(56.5%) | 211(68.3%) | <0.001 |
| Antibiotics | 1980(95.4%) | 305(98.7%) | 0.006 |
| Urine Output before CRRT—ml/24 hours | 13.8(3.8,36.3) | 17.1(4.2,43.8) | 0.060 |
| Fluid balance before CRRT—ml/24 hours | 2020(885,3560) | 2005(750,3447) | 0.869 |
| Coexisting conditions-no. (%) |  |  |  |
| Hypertension | 790(38.1%) | 127(41.1%) | 0.304 |
| Diabetes Mellitus | 748(36%) | 111(35.9%) | 0.970 |
| Hyperlipidemia | 366(17.6%) | 67(21.7%) | 0.085 |
| Coronary artery disease | 474(22.8%) | 72(23.3%) | 0.855 |
| Congestive heart failure | 361(17.4%) | 54(17.5%) | 0.970 |
| Chronic pulmonary disease | 395(19%) | 43(13.9%) | 0.030 |
| Chronic renal disease | 728(35.1%) | 119(38.5%) | 0.238 |
| Malignancy | 409(19.7%) | 40(12.9%) | 0.005 |
| Laboratory data before CRRT |  |  |  |
| Albumin, mg/dL | 2.3(1.8,2.8) | 2.4(1.9,2.9) | 0.137 |
| Hemoglobin, g/dL | 9.7(8.4,11.4) | 10(8.9,11.5) | 0.010 |
| WBC count, 1000/μL | 11.6(7.2,17.6) | 11(7.5,15.5) | 0.184 |
| Platelet count, 1000/μL | 107(58,183) | 104(59,165) | 0.458 |
| pH | 7.3(7.2,7.4) | 7.4(7.3,7.4) | <0.001 |
| Sodium, mmol/L | 138(134,143) | 139(135,144) | 0.142 |
| Lactate, mmol/L | 4.1(1.9,9.1) | 2.7(1.5,5.5) | <0.001 |
| K, mmol/L | 4(3.4,4.7) | 3.9(3.4,4.4) | 0.089 |
| Calcium, mg/Dl | 7.8(7.2,8.4) | 8(7.3,8.5) | 0.102 |
| Base Excess, mmol/L | -7.7(-11.5,-4.5) | -5(-7.7,-2.6) | <0.001 |
| O_2_ Saturation, % | 98.2(95.4,99.6) | 98.4(96,99.8) | 0.174 |
| Creatinine, mg/dL | 2.1(1.3,4) | 1.9(1.3,3.8) | 0.229 |
| Nutritional supplement |  |  |  |
| Total parenteral nutrition | 240(11.6%) | 62(20.1%) | <0.001 |
| Parenteral nutrition | 1593(76.7%) | 200(64.7%) |  |
| None | 243(11.7%) | 47(15.2%) |  |
| Modality of CRRT |  |  |  |
| CVVH | 1873(90.2%) | 281(90.9%) | 0.691 |
| CVVHD | 203(9.8%) | 28(9.1%) |  |
| Outcome |  |  |  |
| RRTFS | 550(26.5%) | 69(22.3%) | 0.119 |
| 72-hour mortality | 789(38%) | 0(0%) | <0.001 |
| 28-day mortality | 1326(63.9%) | 104(33.7%) | <0.001 |
| 90-day mortality | 1453(70%) | 217(70.2%) | 0.933 |

Figure S1. Receiver Operating Characteristic (ROC) analysis to compare the predictive performance of PNI with that of albumin and lymphocytes.


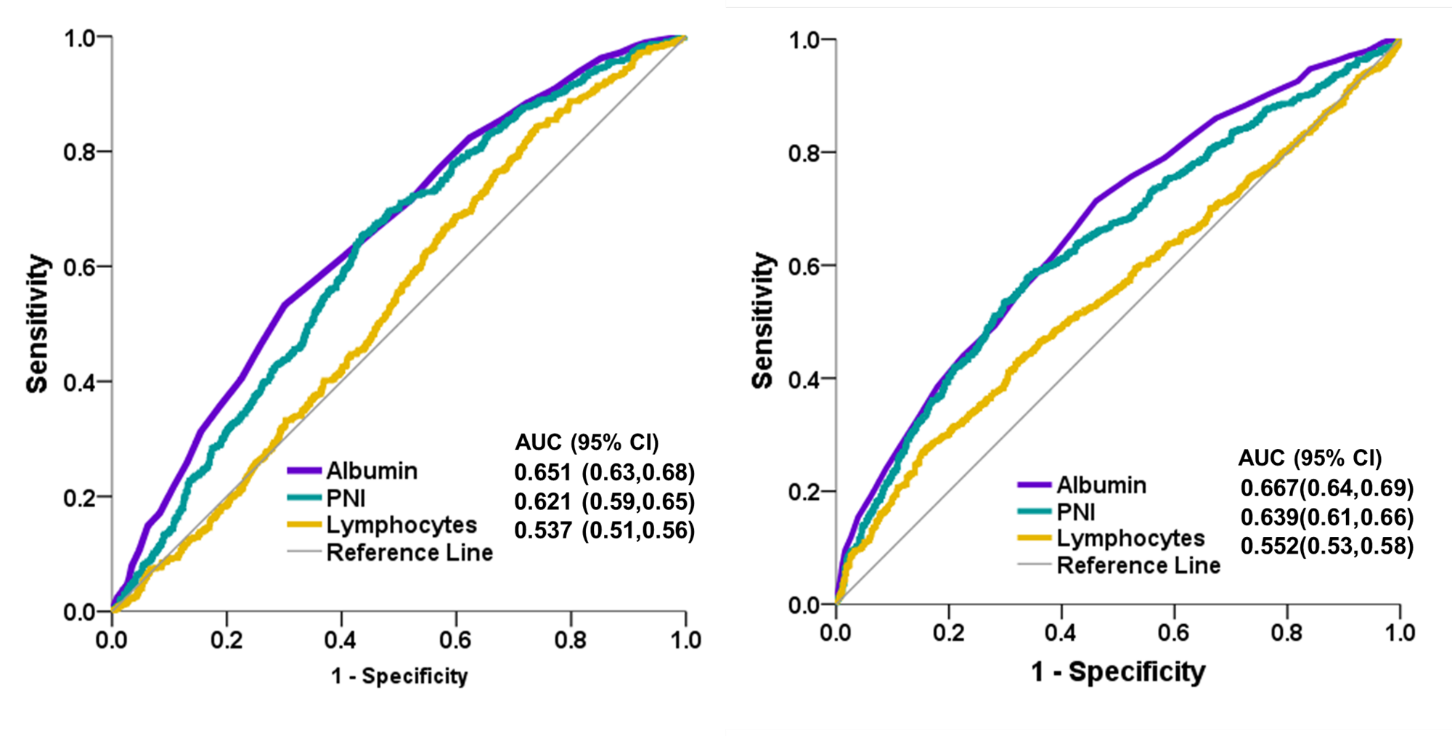


Table S2: The impact of standardized values ​​of nutritional indicators on clinical outcomes:

|  | ***RRTFS*** | | ***72-hr mortality*** | | ***28-day mortality*** | | ***90-day mortality*** | |
| --- | --- | --- | --- | --- | --- | --- | --- | --- |
|  | ***Adj.OR***  ***(95%CI)*** | ***P-value*** | ***Adj. HR  (95%CI)*** | ***P-value*** | ***Adj. HR  (95%CI)*** | ***P-value*** | ***Adj. HR  (95%CI)*** | ***P-value*** |
| Standardized value of PNI | 1.53 (1.20,1.93) | <0.001 | 1.02 (0.89,1.17) | 0.810 | 0.85 (0.76,0.95) | 0.003 | 0.84 (0.75,0.93) | 0.001 |
| Standardized value of albumin | 1.49 (1.26,1.77) | <0.001 | 0.98 (0.89,1.08) | 0.684 | 0.90 (0.83,0.97) | 0.005 | 0.87 (0.81,0.94) | <0.001 |
| Standardized value of lymphocyte | 0.84 (0.57,1.22) | 0.350 | 1.08 (0.91,1.30) | 0.371 | 0.95 (0.81,1.11) | 0.541 | 0.96 (0.83,1.12) | 0.602 |

Nutritional indicators are standardized and transformed to eliminate the influence of units.

Table S3: Hazard ratio (HR) and 95% confidence interval (CI) for 365-day renal outcome according to PNI

|  | ***365-day* dialysis dependency** | | | | |
| --- | --- | --- | --- | --- | --- |
|  | ***Crude HR (95%CI)*** | ***P-value*** | ***Adj. HR (95%CI)*** | ***P-value*** |  |
| Low PNI | 1(reference) |  | 1(reference) |  |  |
| High PNI | 1.02 (0.53,1.97) | 0.953 | 0.98 (0.48,2.03) | 0.961 |  |
